# A novel approach for automatic annotation of human actions in 3D point clouds for flexible collaborative tasks with industrial robots

**DOI:** 10.3389/frobt.2023.1028329

**Published:** 2023-02-15

**Authors:** Sebastian Krusche, Ibrahim Al Naser, Mohamad Bdiwi, Steffen Ihlenfeldt

**Affiliations:** Department of Production System and Factory Automation, Fraunhofer Institute for Machine Tools and Forming Technology, Chemnitz, Germany

**Keywords:** data labeling, human activity recognition, deep learning, robotics, point cloud annotation

## Abstract

Manual annotation for human action recognition with content semantics using 3D Point Cloud (3D-PC) in industrial environments consumes a lot of time and resources. This work aims to recognize, analyze, and model human actions to develop a framework for automatically extracting content semantics. Main Contributions of this work: 1. design a multi-layer structure of various DNN classifiers to detect and extract humans and dynamic objects using 3D-PC preciously, 2. empirical experiments with over 10 subjects for collecting datasets of human actions and activities in one industrial setting, 3. development of an intuitive GUI to verify human actions and its interaction activities with the environment, 4. design and implement a methodology for automatic sequence matching of human actions in 3D-PC. All these procedures are merged in the proposed framework and evaluated in one industrial Use-Case with flexible patch sizes. Comparing the new approach with standard methods has shown that the annotation process can be accelerated by 5.2 times through automation.

## 1 Introduction

Recognition and prediction of human actions are increasingly crucial in industrial production. Flexible and agile machine systems should be able to recognize their environment, detect persons in the workspace and predict human intentions. Based on the future human action information, the machine systems adapt the production sequence in real-time and optimize the production process situationally. Such an activity prediction would allow the human to work very closely with the robot in specific product steps in collaboration without the danger of a collision. On the other hand, there is no loss of machine utilization because the system can increase the speed again when a permissible safety distance is reached (Rashid et al., 2020). By ensuring the safety, interactions such as teaching a heavy-duty robot by gestures very close to the robot can be enabled without requiring complex approvals by the operator The No-Code approach allows the human to teach the robot directly by guiding, showing, or demonstrating without the need for knowledge of complex programming languages (Bdiwi et al., 2016; Halim et al., 2022). Working directly with the robot on the production component enables the human to use his sensitive skills in very complex activities and thus drive the level of automation forward, even in non-industrial areas such as surgery (Su et al., 2021; Su et al., 2022). To make these possible, efficient algorithms are needed, that can robustly recognize and predict human behavior in all its variations. Many approaches have been implemented to deal with different video data (Baradel et al., 2016; Feichtenhofer et al., 2016; Gkioxari et al., 2017; Liang et al., 2019; Morais et al., 2020). Most convert the input video data into spatio-temporal representations and infer labels from these representations. Different types of information are used in these works, such as human posture, interaction with objects, and appearance features.

A large number of published data sets with daily and sports activities are available for the method development (Shahroudy et al., 2016; Kay et al., 2017; Carreira et al., 2018; Carreira et al., 2019; Jang et al., 2020; Liu et al., 2020; Lucas et al., 2020; Shao et al., 2020). Annotating these datasets is very time-consuming and labor-intensive. Human annotators must define and describe spatial regions associated with an image frame from a video or delineate temporal segments in conjunction with the video. Standard shapes such as rectangles, circles, points, or polygons frequently characterize the spatial regions. In contrast, marking the temporal segments requires only the start and end timestamp. These spatial regions and temporal segments are described by textual metadata.

Activities in an industrial context are usually very complex and consist of a combination of simple actions. In most cases, items are used to perform the activity. Up to that, it comes to interactions with other persons to accomplish extended, more complex action sequences. The activity duration is usually longer than 1 s, but the duration of action is mostly only up to 0.5 s (Das Dawn and Shaikh, 2016; Trong et al., 2017; Dang et al., 2021). In very rare cases there are crowds of people or crowded scene, but more often there is occlusion by industrial plant parts and machinery in the scene. Furthermore, many items, such as ladders or chairs, are often classified as humans by 3D sensor systems, depending on their shape. In addition to these static objects, there are dynamic objects such as robots or AGVs, whose position changes continuously, and these temporarily provide occlusions in the workspace.

In order to face these challenges, it is necessary to apply a 3D multi-sensor system that observes the industrial workspace from multiple perspectives and avoids the risk of occlusion. Each 3D sensor provides a 3D point cloud and an RGB image with a frame rate of 10 fps ∼ 30 fps, which leads to a vast amount of data for an action sequence with a duration of about 1s, if at least 4 sensors are used. Manual annotation of action and activity sequences is impossible because of this amount of data and the complexity of such a multi-sensor system. Furthermore, manual annotation of objects in 3D space requires different modeling tools than those required for annotation 2D images.

The automatic annotation approach presented in the paper can fulfill all these requirements. The manual effort of the annotation process would be reduced to a significant amount, and training data from different perspectives can be generated due to the multi-sensor technology. By using deep learning models for skeleton-based recognition of human activities (Pavlakos et al., 2016; Barsoum et al., 2017; Ruiz et al., 2018; Li et al., 2020; Mao et al., 2020; Yuan and Kitani, 2020; Dang et al., 2021; Mao et al., 2021; Martínez-González et al., 2021), the action sequences can be classified and tracked very easily. The annotator no longer needs to focus on the elaborate annotation of the human pose and can take care of tracking multiple people.

In our work, we designed a multi-layered structure of different DNN classifiers to recognize humans and dynamic objects in the 3D point clouds of a multi-sensor system. To do this, we combined several available AI classifiers to distinguish humans from robots or other objects accurately. We developed and implemented a methodology for automatically matching human actions in 3D point clouds for human activity sequence detection. To operate these methods and verify the results, we designed an intuitive user interface that allows the user to correct the automatic annotation or improve the process by optimizing the classifiers. To finally evaluate the approach, we created extensive datasets based on empirical experiments with ten subjects performing various simple and complex activities in an industrial environment. As part of the experiments, we addressed human-robot cooperation scenarios where humans and robots coexist in a workspace very close.

## 2 Related work

Annotation of human activities in video data is very time and labor-intensive work. It requires a massive amount of human and hardware resources. There are two general approaches for generating data sets with human actions.

1) Data sets like NUCLA, SYSU, NTU-RGB + D, PKU-MMD (Bdiwi et al., 2016; Rashid et al., 2020; Su et al., 2021; Halim et al., 2022; Su et al., 2022) (Wang et al., 2014; Shahroudy et al., 2016; Hu et al., 2017; Liu et al., 2017; Liu et al., 2020) were generated under laboratory-like conditions, the activities were controlled, and the sensors had optimal perspectives on the scene. Based on this boundary condition, the human activity in the video sequences can be very well recognized, annotated, and quickly separated. In this case, annotation by hand is very easy and requires less effort. However, human activities' variance is minimal, meaning that the data sets do not represent reality. Performing such predefined laboratory experiments is labor intensive and time-consuming.
2) In contrast, data sets such as Fine-Gym, UAV-Human, HOMAGE (Shao et al., 2020; Li et al., 2021; Rai et al., 2021) generated in real-world environments (such as road traffic and crowds in public places) with uncontrolled action are more challenging to annotate because the environment is too cluttered, people may be obscured, camera perspectives are not optimal, or the variance of human action is too different.

Datasets like as ActivityNet, AVA, Babel (Heilbron et al., 2015; Gu et al., 2018; Punnakkal et al., 2021) have been labeled *via* commercial crowdsourcing platforms such as Amazon Mechanical Turk (AMT) (Amazon, 2021) for a charge to the dataset creators. In some cases, the annotators from crowdsourcing platforms influence the annotation quality negatively due to a lack of expertise. The crowdsourcing method may compromise confidentiality.

There are different open-source tools for annotating objects and features in image videos (da Silva et al., 2020; Dutta and Zisserman, 2019; Biresaw et al., 2016; Riegler, 2014; Yuen et al., 2009). Most of them require a manual annotation by an annotator in every frame. Only some of them provide the ability to track humans or objects across multiple image frames using tracking functions (David, 2000; Vondrick et al., 2012; Bianco et al., 2015; Intel, 2021). This feature makes it easier for annotators to save time by automatically tracking the annotations instead of labeling them frame by frame. Usually, the marking is done by an Annotator manually or by an object recognition algorithm before the tracking function tracks the object or human over several frames. In ViPER, ground truths are stored as sets of descriptors (David, 2000). Each descriptor annotates an associated range of frames by instantiating a set of attributes for that range. However, these attributes are not simple and flexible enough to annotate time-varying (appearing and disappearing) behaviors. VATIC is a simple, reusable, open-source platform for labeling research videos (Vondrick et al., 2012). To annotate human behavior, third parties have extended VATIC with additional features. iVAT (Bianco et al., 2015) presents a tool that allows the user to extract target states and categorize the targets. To significantly minimize human effort, iVAT uses automatic tracking and other computer vision methods combined with interpolation to support manual annotation. However, human interventions and verifications are necessary to validate the quality of the annotation results. JABBA (Kabra et al., 2013) is a semi-automatic machine learning-based behavioral annotator that takes the already annotated states (trajectories) as input to perform the task. The purpose of these tools is to reduce human effort and time and to preserve the annotation quality. Manual labeling effort is reduced by automatically estimating states between selected keyframes using linear interpolation and homography-preserving techniques (Yuen et al., 2009; Vondrick et al., 2012). The annotation quality of these tools depends on the individual annotators or object detectors. It is very challenging to mark objects correctly in crowded scenes, and annotators may easily miss important details. Furthermore, no other information, such as the human body pose or current activity, is provided. In addition, it is necessary to perform a pose estimation to get the skeleton data (Cao et al., 2016; Andriluka et al., 2017; Güler et al., 2018; Kocabas et al., 2018; Xiao et al., 2018; Cai et al., 2019; Cheng et al., 2019; Sun et al., 2019; Jin et al., 2020; Contributors, 2021; Kreiss et al., 2021). The HAVPTAT tool allows the annotation of body poses (“Walking”, “Standing”, “Sitting”) and simple activities (“WalkingWhileCalling”, “StandingWhileWatchingPhone”, “SittingWhileEating”) of several people over a sequence of 2D images (Quan and Bonarini, 2022). A separate algorithm OpenPifPaf (Kreiss et al., 2021), whose results are reloaded and played in parallel with the video, does the estimation of body poses. The annotator has to do the body pose assignment manually.

Besides the above drawbacks, it is challenging to perform the detection and tracking of multiple people in a video when dealing with crowded and cluttered scenes. None of the approaches uses a multi-sensor concept to fuse, and plausible the body pose estimation and tracking, which guarantees that the results can be classified and annotated much more clearly.

## 3 Annotation framework

**FIGURE 1:**
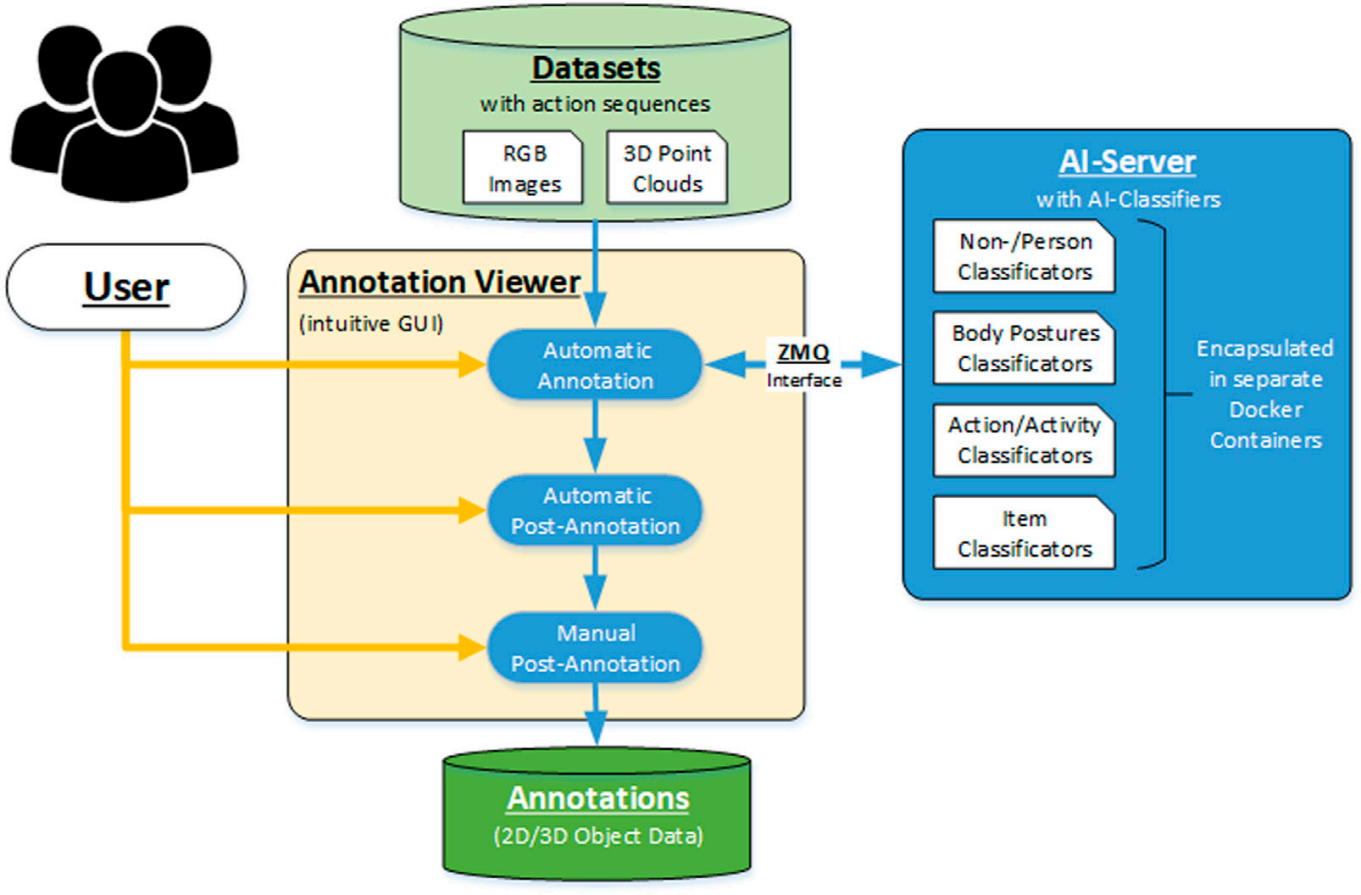
Schematic chart of the annotation framework.

The realization of the annotation approach required the development of an extensive framework, its architecture consisting of a powerful AI server and an intuitive GUI, as seen in Figure 1. The basic workflow is structured into three steps.

1) The user selects the desired action dataset *via* the GUI and passes it to the automatic annotation step, where the data is automatically segmented, tracked, and classified.
2) The raw results are then analyzed, filtered, and optimized in the following automatic post-annotation step. The goal is to determine and correct correlations based on a complete view of the entire sequence of actions.
3) In the final manual post-annotation step, the user checks whether the results are correct or whether a further manual correction is necessary, based on the visualization that displays the annotation results in the context of the 3D point clouds and RGB images.

The development and implementation of this structure were realized using the OpenCV library (Bradski, 2000) for the backend framework, VTK library (Schroeder et al., 2006) for 3D visualization and the Qt library (Nasdaq Helsinki: QTCOM, 2021) for the GUI. The integration of the open-source AI algorithms was done using Python since most approaches are based on AI frameworks PyTorch (Paszke et al., 2019) or Tensorsflow (Abadi et al., 2021), which are implemented in the Python programming language. Each AI classifier is encapsulated in a Docker container to avoid possible inferences between the specific software dependencies. The Docker containers are run on a separate Linux-based AI server with two parallel GPU cards to enhance performance and ensure parallel usage. A ZMQ interface based on UDP is implemented between the Annotation Viewer and Docker Container on the AI Server for data exchange (Hintjens, 2011).

### 3.1 Data structures

Because of the high raw data volume of the multi-sensor system and the output of the multi-person tracking, it is necessary to structure the data so that the system can clearly distinguish between input data and the intermediate and end results. For this purpose, a data structure with specific data types was developed, which lists all data and information types and allows the user to select the available visualization option. The data structure differs in two basic categories, which are specified as follows:

**Streams:** The Streams category contains all image and 3D point cloud data sets of the multi-sensor system for the entire acquisition time of an action sequence, forming the basis for the automated annotation process. One stream includes the acquired sensor data of the scene from the perspective view of the single sensor. The intrinsic and extrinsic calibration parameters of every single sensor are required to establish the relation between the 2D RGB camera images, the 3D point clouds, and the sensor world coordinate system. In addition to the reference to the sensor world coordinate system, the reference to the world coordinate system should also be given for a holistic view of the scene from different perspectives.

**Object List**: This category allows the summary of all objects detected, tracked, and classified throughout the action sequence. Besides the result representation of the automatic annotation process, this structure is also used for the following steps of automatic post- and manual annotation. The list contains a separate data set (object data) for each object, which enables a view of the temporal and spatial movement concerning the whole sequence for the single object. The dataset contains spatial information in the form of 2D and 3D bounding boxes and the results of person/non-person classification and human posture estimation for each frame, respectively. Furthermore, global information about the object is also stored, such as walking paths or the execution location of the action.

**Annotation Data:** For training and verification of AI algorithms for action recognition and action prediction, datasets are required that represent human actions in a temporal context. The annotation sets include point clouds and image patches with corresponding labels related to human action, generated automatically based on object data and specifications by the user.

**FIGURE 2:**
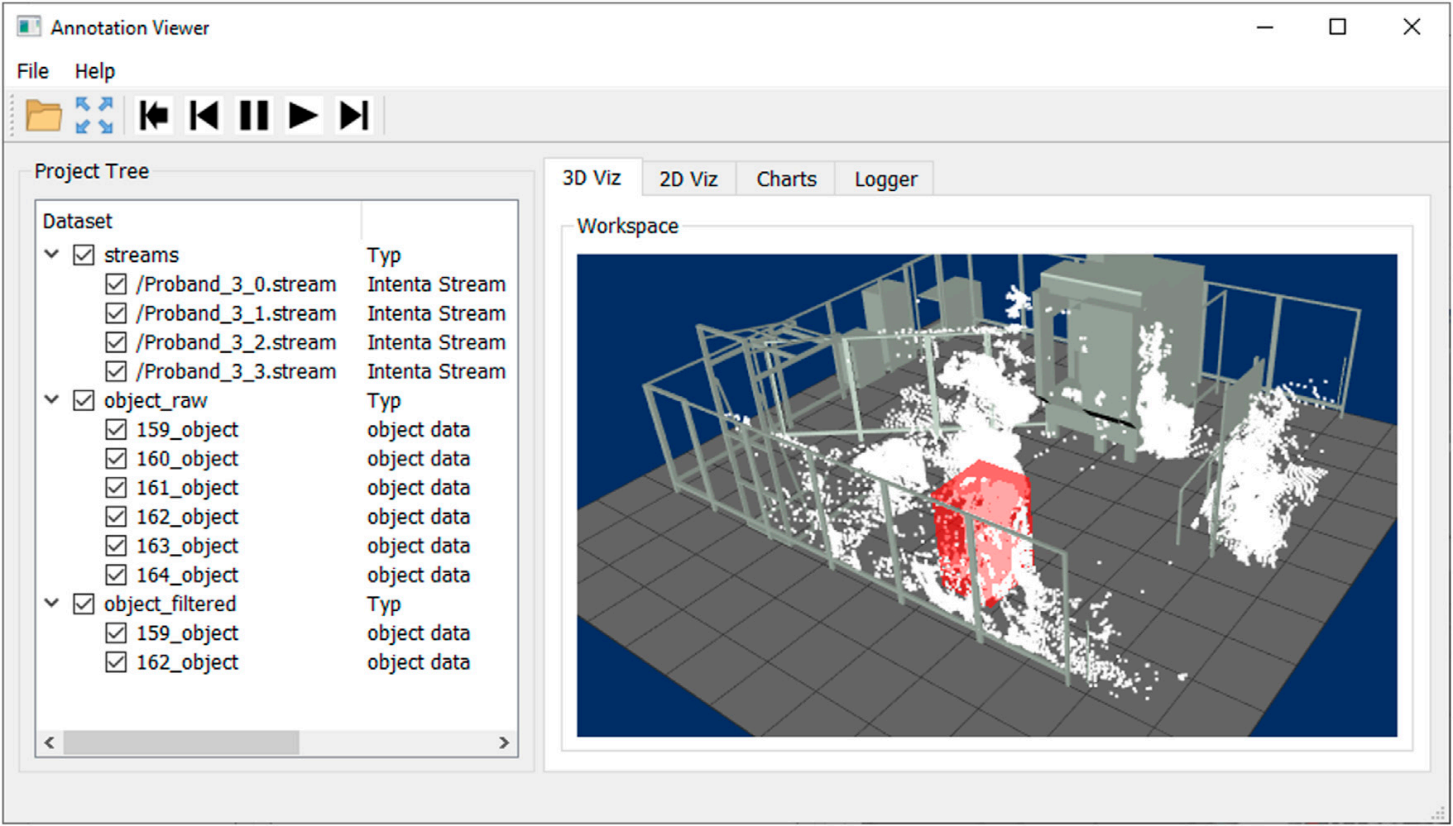
Graphical user interface for displaying sensor data sets and annotation results.

In the user interface, the loaded and generating data structures are visualized in the form of a tree model, shown in Figure 2 on the right side of the user interface. The user can quickly distinguish between raw data (streams), object data (object list), and annotation data based on the structure and select the corresponding visualization forms *via* the checkboxes.

### 3.2 Annotation viewer

An intuitive user interface has been designed and implemented to merge annotations and raw data sets, allowing the user to review, verify and adjust the action sequences. In the interface, the 2D image data, 3D point clouds, and tracking and classification results of an action sequence can be visualized in correspondence to each other frame by frame. Various display forms are implemented as separate visualizations for data representation, defined as follows, as shown in Figure 2.

**FIGURE 3:**
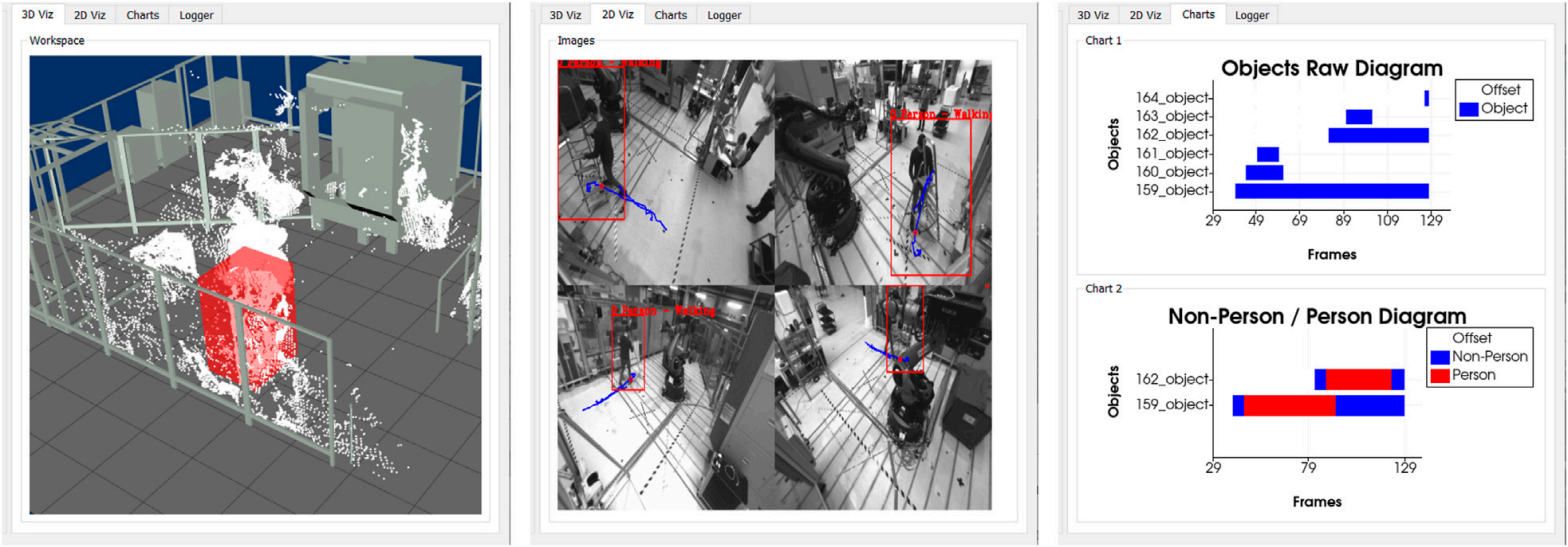
Workspace viewer (left), image viewer (center), chart viewer (right).

**Workspace Viewer:** For displaying the 3D object information of the detected human to the 3D point cloud, a 3D visualization environment is implemented, representing the multi-sensor system surveillance space. The user can load and visualize 3D CAD models of the plant with the machines, robots, and protective fences to reference the sensor information to the environment, as shown in Figure 3 (left). The precondition for correct mapping of the 3D object information, 3D point clouds, and CAD models is a precise extrinsic calibration and temporal synchronization to each other. With the 3D workspace visualization, the user can quickly check and verify the results of the segmentation, tracking, and classification algorithms. In complex scenes with many dynamic objects, the information representation in 3D can be better than in 2D.

**Image Viewer:** Parallel to the 3D workspace visualization, the user can review and analyze the action scene from every single sensor perspective of the multi-sensor system. For this purpose, the RGB images are visualized in combination in a separate tab, as shown in Figure 3 (center). In addition to the perspective view of the scene, the results of the tracking and classification are plotted in the individual images. The detected persons are marked by a 2D bounding box and a label so that the persons can be identified over several frames. Furthermore, additional information about the course of action can be displayed, such as the entire walking path or the location where the action was performed. It is important to note that the detected person may be covered due to the sensor perspective, resulting in an incorrect display.

**Chart Viewer:** To summarize the whole sequence of actions, the temporal series of the objects are displayed in the form of bars on a time axis. The diagrams are available in a third tab, as seen in Figure 3 (right). In the Raw Object chart, all detected objects in the action sequence are displayed, allowing the quality of the segmentation, and tracking to be evaluated. The algorithm could not accurately segment and track the objects over the entire sequence if there are many objects with short time segments. The second chart details the objects by person and non-person. The user can see here which objects can be assigned to the acting persons or interaction objects and which objects are misdetections.

**Project Tree:** In general, to give the user an overview of the data being loaded or generated, the data structure is visualized as a dynamic tree model, as shown in Figure 2 on the right side of the user interface. In addition to the listing of the individual sensor data sets and the detected objects of the action sequence, the data types described in Section 3.1 are also displayed. By using a dynamic tree model, additional data, information, and control elements can be added or adapted very quickly, making the user interface flexible depending on the size and type of the data set.

**Data Logger:** A text browser was placed in an additional tab to display status messages or system information on the annotation process. The user can check which current step the process is or which files have been loaded.

The user can replay the action sequence in its full context through the intuitive user interface or analyze the scene in more detail frame by frame. There are also functions for importing raw data, exporting annotation results, and printing analysis results as diagrams.

### 3.3 Annotation approach

**FIGURE 4:**
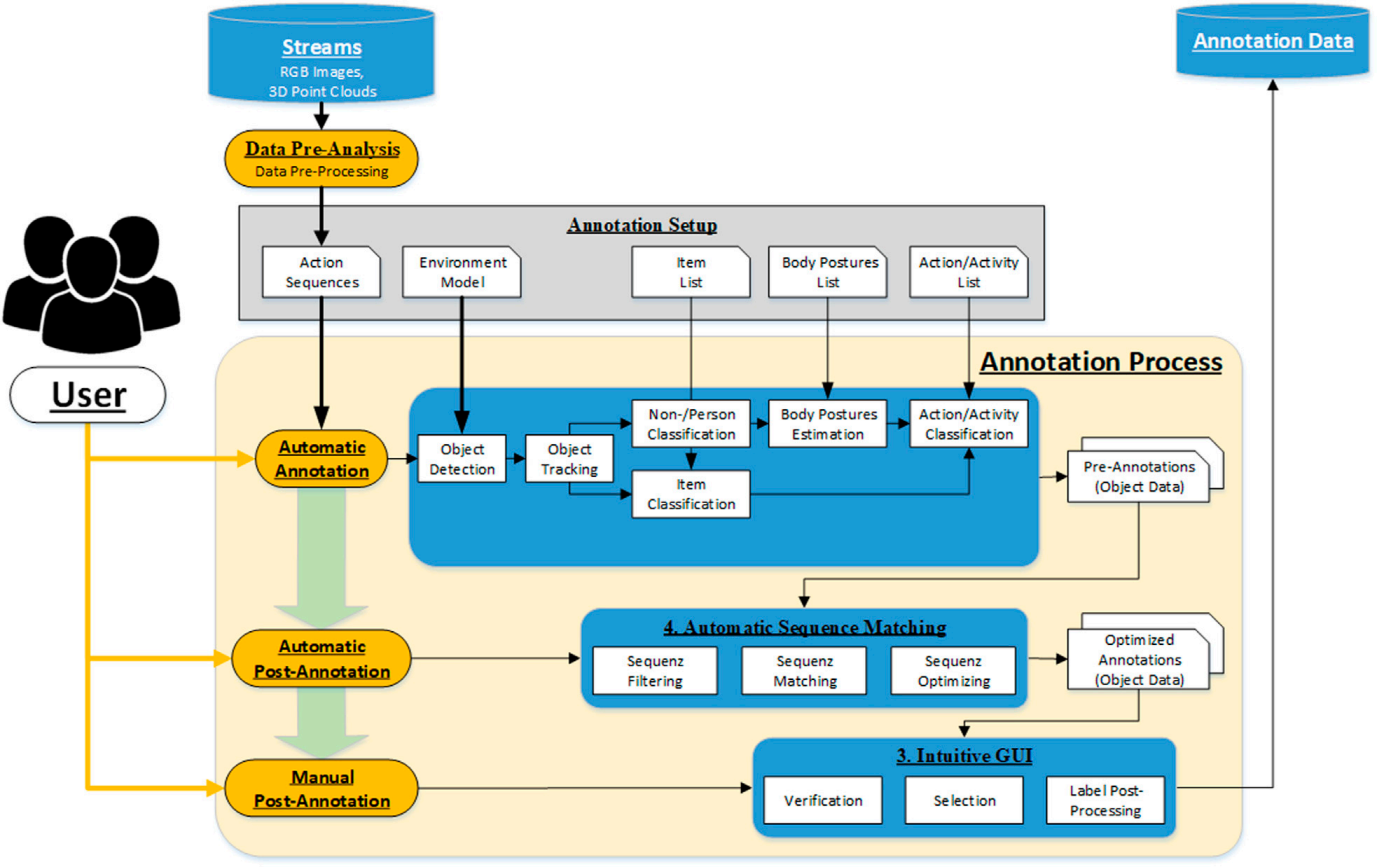
Schematic workflow of the annotation approach.

A workflow approach was developed to ensure that the automatic annotation tool generates action- and context-based annotation data consistent with temporal and spatial relationships. Figure 4 shows the schematic context of the annotation approach. The single steps are explained in detail in the following subsections.

The minimum requirement for the automatic action annotation is that each dataset contains a complete action or a complex activity with a series of actions of at least one person. This action or activity should be represented as a sequence of corresponding RGB images and 3D point clouds in the dataset. A further advantage is if multiple sensors from different spatial perspectives capture the scene with the action sequence time-synchronously. The condition for processing these multi-recordings is that the setup between the sensors is known. The extrinsic parameters must be precisely determined to merge the 3D point clouds of the individual sensors and to ensure the assignment of the detected persons and items. The action sequences must be loaded into the annotation tool as a complete, time-synchronous data set to guarantee successful processing and annotation.

**Data pre-analysis:** The tool treats each annotation of an action dataset as a session, whose results are stored and evaluated separately. After the action sequence is loaded into the annotation tool, a pre-data analysis is performed to check if there is an equal number of RGB images and 3D point cloud or if there is a large number of frame drops. In case of incompleteness or low quality, the data set must be discarded or cropped so that a successive action annotation is possible. In addition, data pre-processing can also be optionally performed, such as the rectification of RGB images or the conversion of depth images from 3D point clouds.

**Annotation Setup:** At the beginning of the annotation session, the user has to provide additional information regarding the action, the environment, and the interaction objects besides the raw data set. The environment model contains all spatial information regarding the action, like where the action takes place, what are possible accesses or walkways in the monitoring area or where are the interaction objects placed, etc. For this purpose, the corresponding objects or regions will be defined using standard shapes such as rectangles, circles, points, or polygons in a predefined XML format. The action inference is based on body posture estimation and object interaction recognition. It is necessary to define the common postures and objects and to submit them as lists to the tool. The final action labeling is done by specifying the action sequence or the action type, which is to be predefined by the user as an action/activity list. In this main list, links are used to refer to the information in the sub-lists. It results in a tree structure with different levels, describing the expected action in detail and forms the basis for the automated annotation algorithm. The user must configure all this information utilizing a parameter catalog and provide it using parameter files.

**FIGURE 5:**
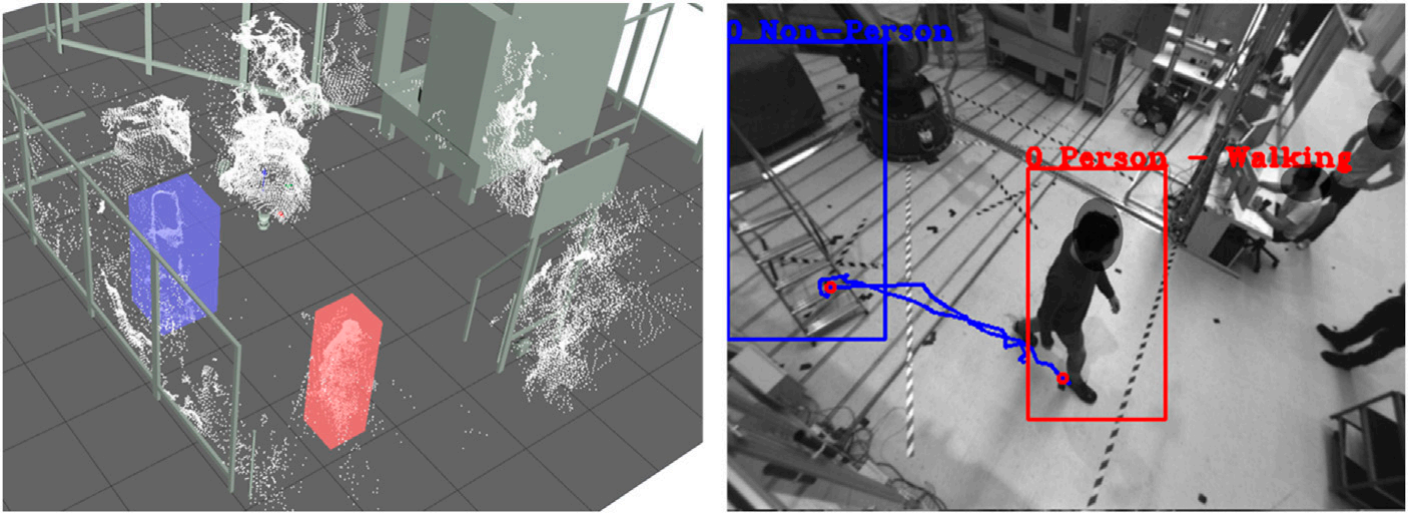
Results of the automatic annotation step visualized in the 3D workspace (left) and RGB image (right) of Sensor 1. People are marked with (red) and objects with (blue).

**Automatic Annotation:** After all boundary conditions regarding the annotation task have been set, and the pre-analysis of the datasets is positive, the datasets can be passed to the automatic annotation step. In the beginning, static objects and environment structures must be removed from the 3D Point Cloud. For this purpose, the background segmentation method is used, which removes all 3D points from the point cloud that are not included in the static background model. This background model should be learned for each scene so that all static objects and environmental structures are precisely removed from the 3D point cloud. A prerequisite is that no dynamic object is in the field of view of the sensors during the teach-in or that there is no further change in the working space of the system. After the segmentation of the static background in the 3D point cloud of the current frame follows the segmentation of all dynamic objects in the sensor’s field of view. All 3D points with a certain Euclidean distance are combined into a cluster, separated from the remaining part of the point cloud by a 3D bounding box, and declared as an object. These segmented objects are then tracked over single frames using Kalman filters until they leave the field of view or the data set with the action sequence is finished. The result of the 3D object segmentation can be seen in Figure 5 (left). Due to background segmentation and point cloud fusion, dynamic objects can be segmented very well from the point cloud.

In order to enable the classification of the segmented objects in the RGB images, it is necessary to project the 3D bounding box from 3D space into the 2D camera plane of the single sensors. For this purpose, the 3D object must be transformed from the world coordinate system into the sensor coordinate system using extrinsic parameters and then projected into the sensor plane using intrinsic sensor parameters. The 3D to 2D projection result can be seen in Figure 5 (right). The object can be segmented from the rest of the RGB image using the 2D bounding boxes. Based on the segmentation, human and object classification in 3D/2D is feasible. The extracted 3D and 2D patches are transferred to the AI server *via* the ZMQ interface. Various AI classifiers such as OpenPose (Cao et al., 2018), Alpha Pose (Li et al., 2018), or DarkNet (Redmon and Farhadi, 2018) can distinguish non-persons from persons or identify specific items. Once a person has been confidently classified, human body pose estimation is performed based on the skeleton model of the OpenPose and AlphaPose classifiers. With the help of the estimator, human postures such as walking, standing, sitting, bending, and kneeling can be detected and additionally used later to generate action-related annotation data.

**FIGURE 6:**
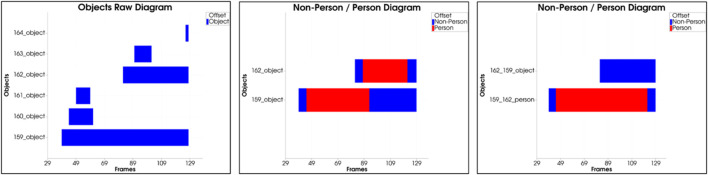
(left) Input: distribution of raw objects over the entire sequence, (center) Filtering: removal of all small undefined objects, (right) Optimization: object decompensation and reordering by Non-Person/Person.

**Automatic Post-Annotation:** The objective of the automatic post-annotation is to view the results of the automatic annotation step over the entire sequence of actions and the overall workspace. Input is the classified objects whose distribution over all sequence frames can be seen in the form of a bar chart in Figure 6 (left). Mainly there are two large objects with a history spanning several frames, from which the plot can be derived. All other objects are short-lived and have been classified as undefined. The appearance can be reduced to artifacts in the point cloud, which are caused by an asynchrony of the sensor data.

The second step of Automatic Post-Annotation includes filtering objects according to different criteria. Besides undefined objects and objects with a small lifetime, all objects with small sizes are removed. The threshold value here is set to a length of 0.3 m per bounding box edge. The object is filtered out if all three edges are below this threshold. The result of the filtering is Figure 6 (center). All undefined objects with a too short lifetime were removed.

After the list of objects has been roughly filtered, the single objects are analyzed in detail. It is examined whether the classification results of the objects are constant over the entire sequence or whether there is a relationship between them. Besides the classification results, the motion sequence in 3D space and the object’s volume is another input for detailed investigation. In the present case, the second object results from the first object, only that the class assignment is incorrect. To correct this, both objects are decompensated in the third stage, rearranged, and reassembled. The result is shown Figure 6 (right). The reordering shows that both object courses are consistent, and a clear distinction between non-person and person can be made.

**Manual Post-Annotation:** Finally, after the object data has been generated and optimized, the final step is verifying and selecting the annotation data to be exported. The user reviews the results over the entire sequence of actions using the 3D Workspace Viewer, 2D Image Viewer, and Chart Viewer by replaying the data or examining it frame by frame. Objects can be manually removed if it becomes apparent that the data has been incorrectly segmented, classified, and mapped. Furthermore, in case of inaccurate results, it is desirable to adjust the segmentation, tracking, or classification parameters and repeat the automatic annotation process.

The automatic annotation approach aims to generate specific action and contextual object data whose spatial and temporal changes are coherent. It ensures that the segmented, tracked, and classified objects are based on the 3D/2D sensor data corresponding to the natural dynamic objects represented by an object or a person. The user can automatically create training and verification datasets based on consistent object data for AI algorithm development. For that purpose, the tool automatically extracts the point cloud or image patches of the selected object or person with the corresponding label using the 3D and 2D bounding boxes.

## 4 Experiments

### 4.1 Experiment setup

The performance of the annotation tool was examined and verified in 6 specific test scenarios according to different criteria. In these scenarios, a test person performs different complex activities ranging from actions such as walking with an object to interactions between two people. The test scenarios cover expected human behaviors in industrial activities in the automotive industry. One of the first actions in the event of malfunctions in industrial robot systems is usually for the worker to enter the robot cells to clear a fault. Therefore, almost every test scenario includes walking with or without an object.

Table 1 summarizes the 6 scenarios with the main actions and the number of persons. The test scenarios range from very simple to complex. Depending on the sequence, one or more persons are in the robot cell, interacting with objects such as ladders, suitcases, or transport carts. The test scenarios cover expected human behaviors in industrial activities in the automotive industry. One of the first actions in the event of malfunctions in industrial robot systems is usually for the worker to enter the robot cells to clear a fault. Therefore, almost every test scenario includes walking with or without an object.

**TABLE 1:** Test scenarios to investigate the performance of the annotation tool.

Scenario-Nr.:	Scenario title	Action types	Active subjects
1	Person walks into robot cell	Standing (static), walking	1
2	Person walks with item	Standing (static), walking, setting up ladder	1
3	Person pushes a transport cart	Standing (static), walking, pushing transport cart	1
4	2 persons walk into robot cell	Standing (static), walking	2
5	2 persons hand over an item	Standing (static), walking, handing over item	2
6	2 persons with a transport cart	Standing (static), walking, Pushing transport cart	2

**FIGURE 7:**
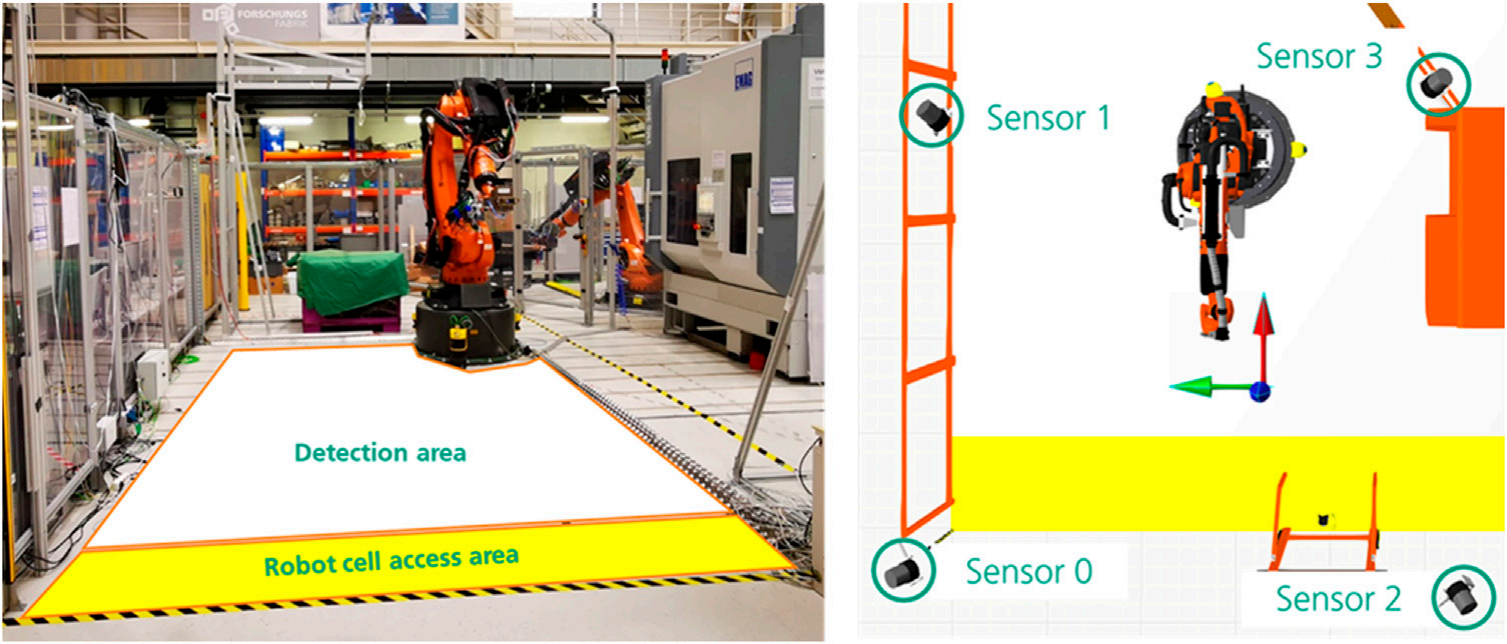
(Left) Front view of HRC test cell at Fraunhofer IWU, (Right) Sensor layout with 4 sensors.

The sensor data is collected in the HRC cell at Fraunhofer IWU, whose design corresponds to a robot cell without a protective fence in the industrial production environment. The interior of the cell is large and barrier-free and allows human activities and interactions in the robot environment. The open area of the cell allows for the optimal alignment of sensor technology to the scenery and a promising field of view without being obscured by additional machine or plant parts. Based on the described infrastructure, the access area and the front working area of the cell were selected for data acquisition of the multi-sensor system. The front view of the HRC cell can be seen in Figure 7 (Left). In order to completely cover the workspace and to avoid obscuring the person acting, four sensors were installed in the room, which records the scene from various perspectives. The sensor layout can be seen in Figure 7 (right).

In the execution of the experiment, data sets with 10 different test subjects with five scenarios each were created based on the test description. Because three scenarios were performed in cooperation with another subject, it is possible that in some data sets, the same subject interacted several times or that the test series of some subjects were combined. The data sets were divided into single- and multi-subject groups for further processing and analysis. Because of the experimental scenarios with two subjects, the number of data sets in both groups is not the same. The recordings resulted in 31 data sets for single-person and 27 for multi-person scenarios.

### 4.2 Statistical assessment

**FIGURE 8:**
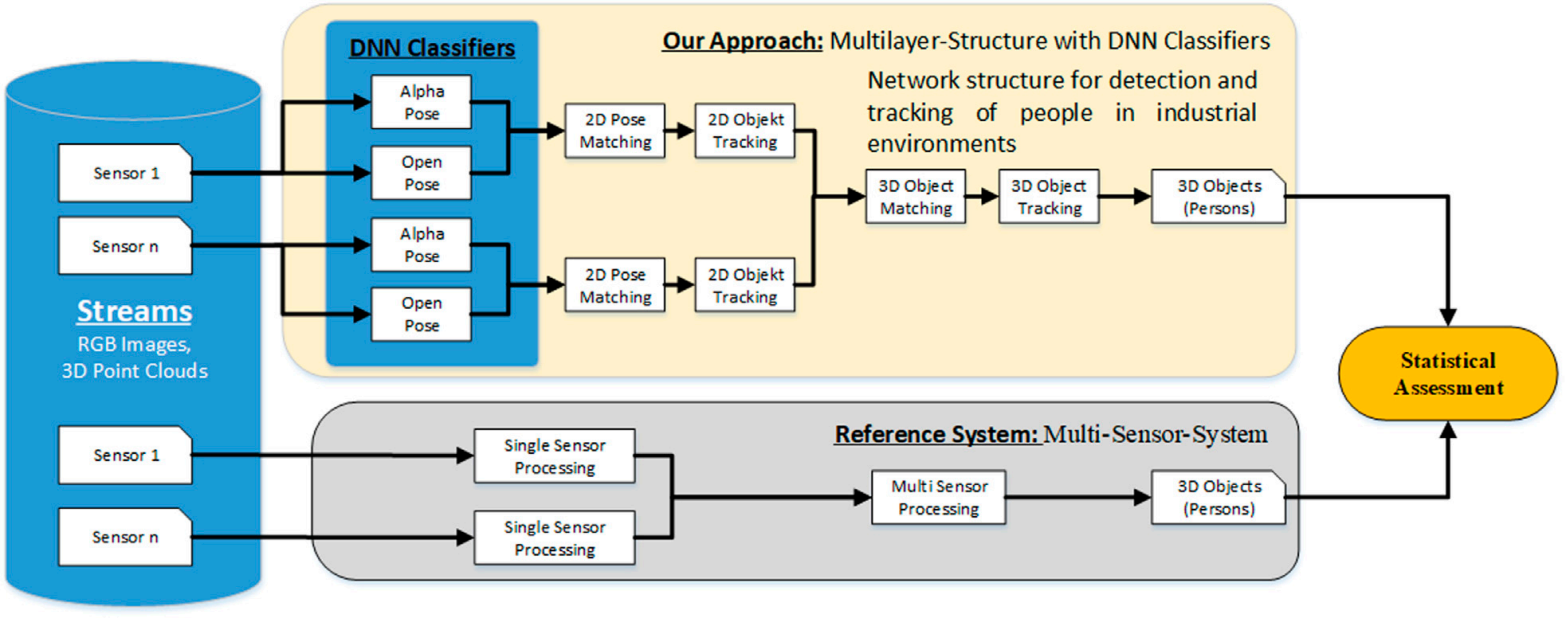
Architecture for comparing Results of Annotation Tool and Reference System.

Based on the data collection, an extensive static study was conducted to demonstrate the performance of the automatic annotation tool. The purpose of this evaluation was to determine how accurately the Multilayer Structure of the various DNN classifiers can classify and track human subjects in 2D and 3D. For comparison, result data from a Multi-Sensor Reference System was used, which provides similar results in 3D space. The Architecture used for the evaluation is shown in Figure 8. The reference system works with the same sensor data as the automatic labeling tool but is limited in its function, the evaluation point cloud data. The recognition of additional objects is not provided, which is necessary for the further development and annotation of more complex scenarios. Therefore, only the recognition of persons is used to compare the systems.

The execution of the tests was automated so that the data sets were replayed. Based on the image data and point clouds, the algorithms classified and tracked people and objects in parallel. Each frame counted the number of objects for each processing step, and the intermediate results were stored.

Supplementary Appendix S1 shows the complete evaluation for scenario 1 with all four sensors. For traceability, the entire process was broken down into individual processing steps, and the number of input and output data was listed for each step. Each row of the table represents the summarized evaluation of a data set. The individual cells represent the cumulative number of input and output data of a processing step. The unit of the cell values is the number of objects processed during the data set’s application. At the table’s end, each column’s mean and median is calculated for comparison against the other scenarios.

Supplementary Appendix S2 summarizes the results of the separate evaluation of all six scenarios and is directly compared with the reference system. In the beginning, average values of the individual processing steps for each sensor are listed, which are then merged in the multi-sensor fusion part. Based on this compact representation, anomalies and high error rates of the individual processing steps can be detected depending on the complexity of the scenario.

The results of the automatic annotation tool and the multi-sensor reference system for each scenario were summarized in Table 2 for the final evaluation of the statistical assessment. The values in the cells are the average number of processed objects. The crucial columns (marked in green) for comparing the systems reflect the number of detected persons. The average values show no significant large differences between the systems. In the case of the more complex scenarios, the number of detected persons is higher for the entire scenario, which is not necessarily due to incorrect classification. Instead, these differences can be attributed to tracking errors or random persons at the edge of the test environment, such as the recording supervisor. For a further analysis of the error causes, the data sets must be looked through randomly. For this purpose, various functions for replaying the data sets are provided in the GUI of the annotation tool. The qualitative assessment section will provide a detailed description of the classification and tracking errors.

**TABLE 2:** Final summary of the statistical evaluation.

Dataset information					3D person tracking (average number of processed objects)			Reference (average number of processed objects)			
Scenario Number	Number of Datasets	Scenario Mode	Number Subjects	Frames	Total	Valid (Person)	Invalid	Raw	Filtered	Person	Non Person
Scenario 1	11	single	1	167	4,0	1.5	2.5	1.5	1.1	1.1	0.0
Scenario 2	10	single	1	182	3.9	1.4	2.5	4.8	1.8	1.4	0.4
Scenario 3	10	single	1	236	8.4	1.9	6.5	5.3	2.0	0.7	1.3
Scenario 4	9	multi	2	179	8.6	2.9	5.7	2.8	2.2	2.2	0.0
Scenario 5	8	multi	2	182	10.4	3.5	6.9	4.8	2.5	2.3	0.3
Scenario 6	8	multi	2	259	13.8	3.8	10.0	7.9	3.3	1.8	1.6

### 4.3 Qualitative assessment

The recorded data sets were then processed with the developed annotation framework. Based on the results, an initial qualitative assessment of performance can be made. Basically, it can generally be concluded that the distinction between non-person and person is accurate in 90% of the scenarios. However, in the case of more complex actions, this leads to inaccurate tracking and classification results. Table 3 summarizes the most significant assessment for the corresponding scenario.

**TABLE 3:** Summary of the qualitative assessment.

Scenario-Nr.:	Scenario title	Segmentation and tracking	Classification
1	Person walks into robot cell	Segmentation and Tracking is correct	Classification is correct
2	Person walks with item	Object ID of human changes to ladder and human is recognized as new object (Figure 9)	Ladder is recognized as a person if the bounding box includes the person (Figure 10)
3	Person pushes a transport cart	Person is not detected in the point cloud (Figure 11)	Person is classified as non-person because only the transport cart is segmented (Figure 11)
4	2 persons walk into robot cell	Segmentation and Tracking is correct	Classification is correct
5	2 persons hand over an item	Segmentation and Tracking is correct	Classification is correct
6	2 persons with a transport cart	Change of object IDs when turning and handing over the transport cart	Transport cart is recognized as a person if the bounding box includes the person

**FIGURE 9:**
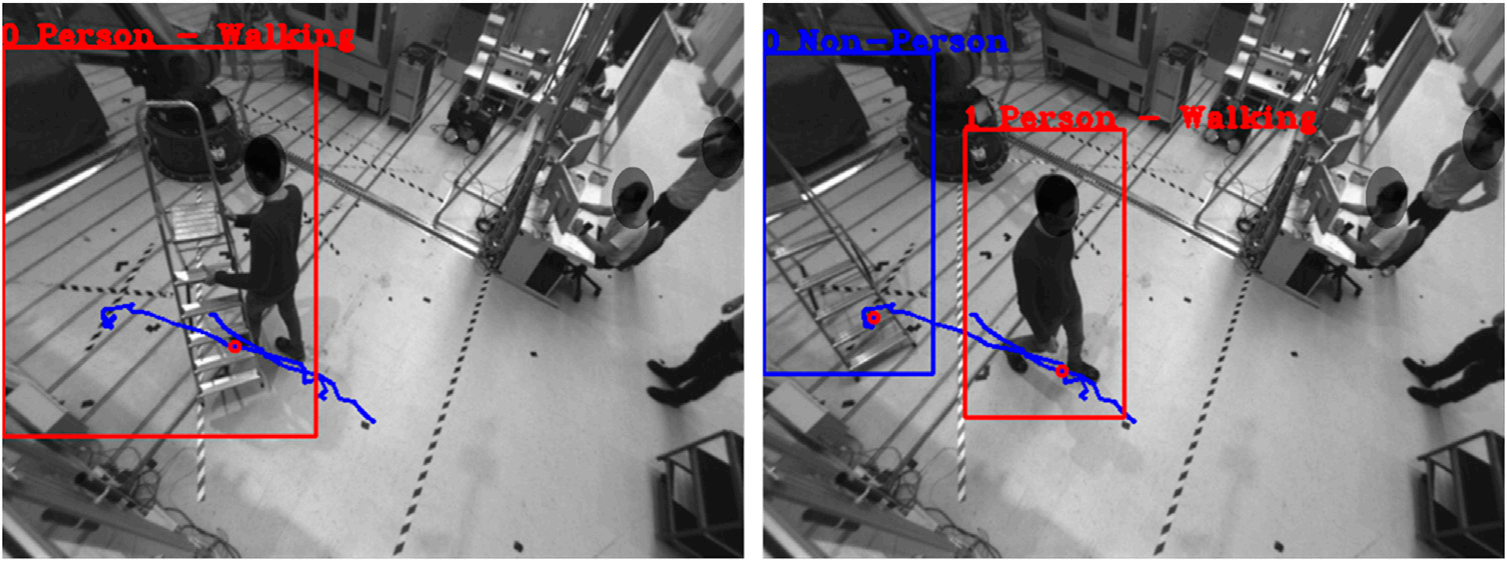
Object ID of human changes to ladder and human is recognized as new object: (Left) human and ladder interact before ID change, (Right) human and ladder separate after ID change.

**FIGURE 10:**
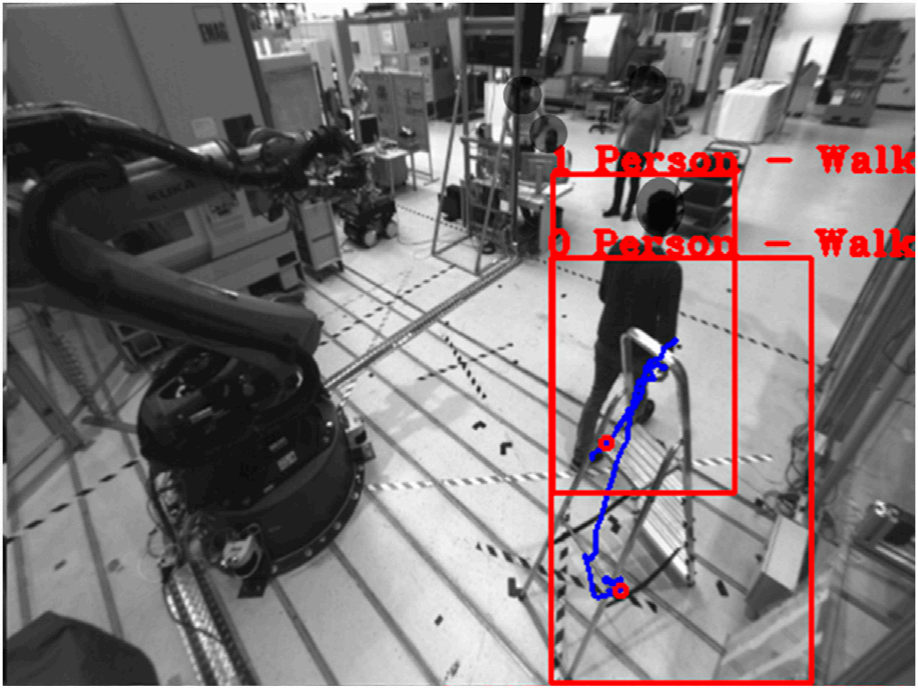
Ladder is recognized as a person if the bounding box includes the person.

**FIGURE 11:**
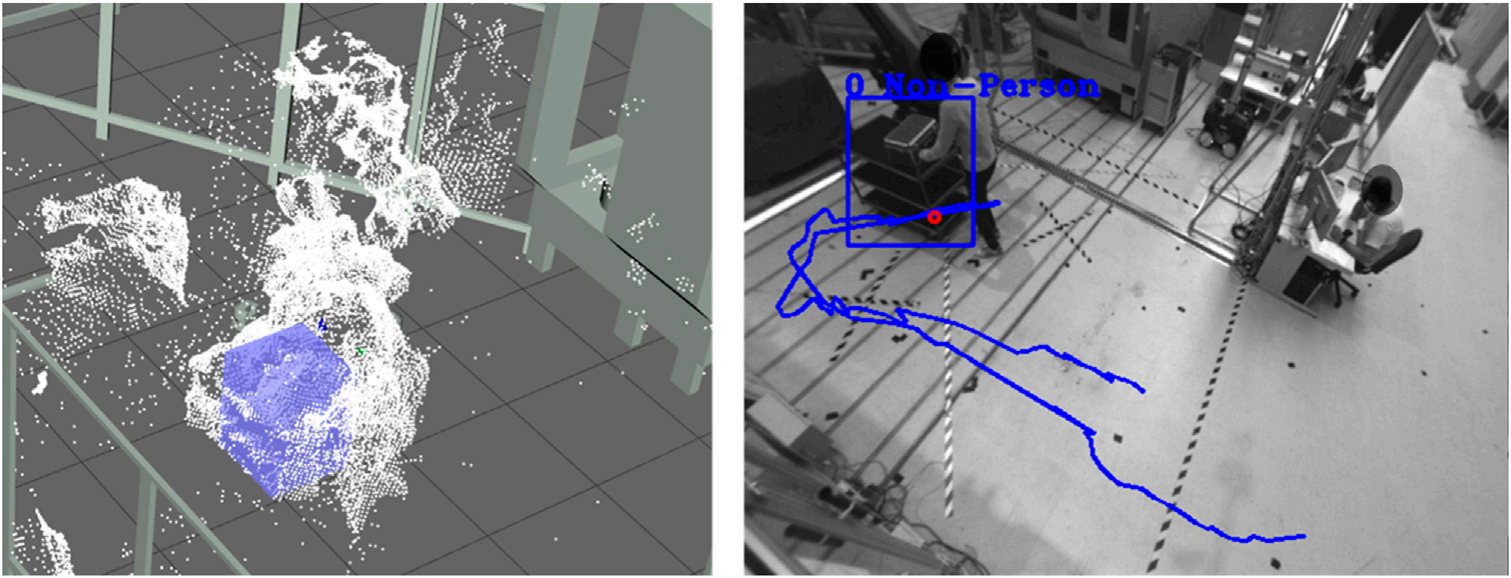
(Left) undetected Person in point cloud, (Right) unclassified Person in RGB Image.

### 4.4 Manual annotation vs. automatic annotation

For a direct comparison of the automatic annotation *versus* manual annotation, the elapsed times of the individual processing steps during the automatic run were recorded and accumulated for all data sets. Table 4 summarizes the results according to the scenarios. The values from the columns for 2D Pose Estimation refer to the processing of the entire image by the pose classifier because the time is independent of the number of objects. The remaining values in the table always refer to the elapsed time per automatically annotated object. Table 4 shows that the classification of the human pose takes the most time. The times per image are about 84 ms for OpenPose and about 94 ms for AlphaPose. In contrast, matching the 2D poses takes only a short time of about 2 ms per object. The projection of the 2D results into the 3D space requires an average time of 17–35 ms. The reason for this is the additional object segmentation in the 3D point cloud, which lead to different times depending on the object’s size. Similar to matching the 2D poses, the 3D object tracking requires a short time of about 3 ms. In total, the average elapsed time per object is 207 ms.

**TABLE 4:** Summary of the average time measured for each processing step per scenario.

Dataset information					Elapsed time in ms					
					2D pose estimation		2D person matching	3D projection	3D object fusion and tracking	Total time
Scenario number	Number of datasets	Scenario mode	Number subjects	Frames	Open pose	Alpha pose				
Scenario 1	11	single	1	167	84.0	92.9	0.9	25.6	2.3	205.6
Scenario 2	10	single	1	182	83.9	92.7	1.3	37.2	3.1	218.2
Scenario 3	10	single	1	236	84.0	93.2	1.4	30.9	3.0	212.5
Scenario 4	9	multi	2	179	83.7	95.4	1.4	18.7	5.4	204.7
Scenario 5	8	multi	2	182	83.8	95.6	1.1	17.5	3.1	201.1
Scenario 6	8	multi	2	259	83.9	95.6	1.2	19.4	3.1	203.2
									Mean	**207,6**

Bold values are the average time over all scenarios.

We use previously confirmed results from standard methods for labeling images with object bounding boxes (Russakovsky et al., 2014; Kuznetsova et al., 2020) or outlines (Lin et al., 2014) to evaluate manual annotation, which is typically done in two steps. In the first stage, annotators are asked to mark the presence or absence of object classes in each image. In the second stage, the annotators draw 2D bounding boxes corresponding to the class labels in the image to segment the object. Another approach to fast annotation uses speech and mouse interaction. By combining them, the annotator can simultaneously draw a bounding box around the object and specify its class by speech (Gygli and Ferrari, 2020). A qualitative comparison is shown in Table 5 to estimate how efficient the automatic annotation approach is. The values for both standard approaches were taken from the existing publication (Gygli and Ferrari, 2020) and were not quantified in an experiment. For the estimation of the manual verification, as provided in the approach, a time of 2.2 s was chosen, which was taken from the publication (Papadopoulos et al., 2016). The proposed automatic annotation approach can be estimated to be x5.2 faster in providing the class and bounding box, including human verification, than the two-stage approach.

**TABLE 5:** Qualitative comparison of the presented automatic annotation approach with standard methods.

	Two-stage approach (Gygli and Ferrari, 2020)	Box & speak (Gygli and Ferrari, 2020)	Ours (DNN classifiers + human verification (Papadopoulos et al., 2016))
Time/box	12.5 s	6.5 s	2.4 s (0.207 s + 2.2 s)
Acceleration of our approach compared to standard methods	x5,2	x2,7	-

## 5 Conclusion

We introduced the automatic annotation framework, an approach capable of cost-effectively generating high-quality annotations for 3D multi-sensor datasets with complex action sequences.

1) Our work focused on designing a multi-layer structure with various DNN classifiers to detect humans and dynamic objects using 3D point clouds. The action sequences can be classified and tracked very easily by using deep learning models for skeleton-based human activity recognition. The annotator no longer needs to focus on the complex annotation of the human pose and can take care of tracking multiple people.
2) The empirical experiments with more than 10 subjects to capture datasets of human actions and activities in an industrial environment allowed us to have a reasonable basis for developing and verifying the whole annotation framework. The various complex scenarios allowed us to specify the requirements for the annotation tool very well.
3) By developing an intuitive graphical user interface (GUI), the user gets a tool to verify and correct the results of the automated annotation process. The annotated action sequences can be referenced over the entire sequence or frame by frame using various 3D and 2D visualizations.
4) The design and implementation of a methodology for automatic matching of human actions in 3D point clouds enable the automatic correction of tracking and classification errors resulting from the multi-layer structure. The decompensation and rearrangement by non-person/person ensure that 3D objects are consistent.

A limitation of the approach is the presence of dynamic, non-human objects such as robots or AGVs, which may need to be clarified parts of the scene or lead to incorrect recognition. Implementing additional AI classifiers or creating a complex kinematic model for contextualization is necessary, especially when annotating human-robot cooperation scenarios where humans and robots work very closely together. This weakness needs to be compensated in the future by using robotic AI classifiers that extract accurately from the scene. To make the approach robust against the described errors in Section 4.3. Qualitative Assessment, several optimizations and tunings are required, which are prioritized as follows.

1) 3D point cloud segmentation: This requires accurately examining the sensor data for errors such as missing 3D points or adjusting segmentation parameters to ensure the segmentation of finer objects.
2) Tracking behavior: In addition to segmentation results, classification results such as human body pose should also be included in tracking to ensure that objects from the previous frame are correctly assigned to the current frame.
3) Multi-layer structure: by using additional DNN classifiers, additional object features should be detected, such as whether the person is carrying or holding an item.
4) Methodology for automatic sequence matching: In addition to the classification results, the interaction with the environment should also be considered, e.g., whether the action is performed at a specific location.

By using the tool, records from multi-sensor systems can be processed synchronously to detect and track the activity of acting individuals seamlessly. Observation from multiple perspectives creates the advantage of having sufficient samples of the human from various views in the sets of annotation data, ensuring that the AI being trained covers a high variance of human behavior. By using multi-modal data such as RGB images and point clouds from multiple sensors, a larger workspace can be covered, and tracking of multiple people can be guaranteed throughout the activity. Especially in human-robot cooperation, where safety has to be ensured during direct interaction in very confined spaces, annotating multimodal sensor data observing a scene from multiple perspectives can lead to a significant optimization of the database for training AI classifiers. Furthermore, through fusion and synchronization, annotation of the multi-modal data in 2D and 2D is possible. The annotation framework was developed to speed up the process of annotating action records and reduce the manual task of the annotator. The proposed approach can accelerate the process by up to x5.2 through automation. The tool is intended to shift the focus from viewing single images to viewing the whole scenario and include the interaction with the environment during the action. This paper is intended to stimulate the creation of more large action datasets and lead to innovations in data-driven computer vision in the coming years.

## Data Availability

The original contributions presented in the study are included in the article/Supplementary Material, further inquiries can be directed to the corresponding author.
