# Supplementary material for: A novel approach for automatic annotation of human actions in 3D point clouds for flexible collaborative tasks with industrial robots
Source: Front Robot AI. 2023 Feb 15;10:1028329. doi: 10.3389/frobt.2023.1028329 (PMC9975387; doi:10.3389/frobt.2023.1028329)
Supplement: Supplementary file 1 [file DataSheet1.docx]

# APPENDIX I - Statistical Evaluation of all Data Sets of Scenario 1

Table 6 Results Summary 2D Sensor Data Processing - Scenario 1 for Sensor 1*

| Dataset Information | | | | | Sensor 1 | | | | | | | |
| --- | --- | --- | --- | --- | --- | --- | --- | --- | --- | --- | --- | --- |
|  |  |  |  |  | 2D Pose Estimation | | 2D Person Matching | | | 2D Person Tracking | | |
| Dataset Number | Scenario Number | Scenario Mode | Number Subjects | Frames | Open Pose | Alpha Pose | Matched | Unmachted OpenPose | Unmatched AlphaPose | Total | Valid | Invalid |
|  | 1 | single | 1 | 178 | 533 | 671 | 475 | 57 | 196 | 13 | 9 | 4 |
|  | 1 | single | 1 | 147 | 292 | 409 | 272 | 20 | 137 | 3 | 2 | 1 |
|  | 1 | single | 1 | 144 | 305 | 405 | 258 | 47 | 147 | 6 | 3 | 3 |
|  | 1 | single | 1 | 144 | 275 | 401 | 263 | 11 | 139 | 2 | 2 | 0 |
|  | 1 | single | 1 | 192 | 301 | 479 | 284 | 17 | 195 | 2 | 2 | 0 |
|  | 1 | single | 1 | 171 | 333 | 510 | 331 | 2 | 179 | 2 | 2 | 0 |
|  | 1 | single | 1 | 209 | 213 | 317 | 208 | 5 | 109 | 1 | 1 | 0 |
|  | 1 | single | 1 | 171 | 338 | 500 | 326 | 12 | 174 | 2 | 2 | 0 |
|  | 1 | single | 1 | 153 | 289 | 451 | 283 | 6 | 169 | 2 | 2 | 0 |
|  | 1 | single | 1 | 139 | 216 | 391 | 206 | 10 | 185 | 8 | 2 | 6 |
|  | 1 | single | 1 | 184 | 343 | 515 | 319 | 24 | 196 | 3 | 3 | 0 |
|  |  | Median Values: | | 171 | 301 | 451 | 283,0 | 12,0 | 174,0 | 2,0 | 2,0 | 0,0 |
|  |  | Mean Value: | | 167 | 312,55 | 459 | 293,2 | 19,2 | 166,0 | 4,0 | 2,7 | 1,3 |

Table 7 Results Summary 2D Sensor Data Processing - Scenario 1 for Sensor 2*

| Dataset Information | | | | | Sensor 2 | | | | | | | |
| --- | --- | --- | --- | --- | --- | --- | --- | --- | --- | --- | --- | --- |
|  |  |  |  |  | 2D Pose Estimation | | 2D Person Matching | | | 2D Person Tracking | | |
| Dataset Number | Scenario Number | Scenario Mode | Number Subjects | Frames | Open Pose | Alpha Pose | Matched | Unmachted OpenPose | Unmatched AlphaPose | Total | Valid | Invalid |
|  | 1 | single | 1 | 178 | 266 | 443 | 263 | 3 | 180 | 11 | 5 | 6 |
|  | 1 | single | 1 | 147 | 221 | 329 | 213 | 8 | 116 | 18 | 5 | 13 |
|  | 1 | single | 1 | 144 | 271 | 536 | 261 | 10 | 273 | 4 | 4 | 0 |
|  | 1 | single | 1 | 144 | 288 | 400 | 270 | 18 | 130 | 2 | 2 | 0 |
|  | 1 | single | 1 | 192 | 274 | 331 | 241 | 33 | 89 | 11 | 4 | 7 |
|  | 1 | single | 1 | 171 | 202 | 331 | 177 | 25 | 154 | 6 | 1 | 5 |
|  | 1 | single | 1 | 209 | 183 | 250 | 181 | 2 | 70 | 1 | 1 | 0 |
|  | 1 | single | 1 | 171 | 305 | 486 | 301 | 4 | 185 | 3 | 2 | 1 |
|  | 1 | single | 1 | 153 | 286 | 305 | 261 | 25 | 44 | 7 | 3 | 4 |
|  | 1 | single | 1 | 139 | 276 | 367 | 262 | 14 | 106 | 3 | 2 | 1 |
|  | 1 | single | 1 | 184 | 205 | 266 | 200 | 5 | 66 | 6 | 2 | 4 |
|  |  | Median Values: | | 171 | 271 | 331 | 261,0 | 10,0 | 116,0 | 6,0 | 2,0 | 4,0 |
|  |  | Mean Value: | | 167 | 252,45 | 367,64 | 239,1 | 13,4 | 128,5 | 6,5 | 2,8 | 3,7 |

* Each row of the table represents the summarized evaluation of a data set. The individual cells represent the cumulative number of input and output data of a processing step. The unit of the cell values is the number of objects processed during the data set's application. At the end of the table, the mean and average of each column are calculated for comparison against the other scenarios.

Table 8 Results Summary 2D Sensor Data Processing - Scenario 1 for Sensor 3

| Dataset Information | | | | | Sensor 3 | | | | | | | |
| --- | --- | --- | --- | --- | --- | --- | --- | --- | --- | --- | --- | --- |
|  |  |  |  |  | 2D Pose Estimation | | 2D Person Matching | | | 2D Person Tracking | | |
| Dataset Number | Scenario Number | Scenario Mode | Number Subjects | Frames | Open Pose | Alpha Pose | Matched | Unmachted OpenPose | Unmatched AlphaPose | Total | Valid | Invalid |
|  | 1 | single | 1 | 178 | 100 | 186 | 93 | 7 | 93 | 1 | 1 | 0 |
|  | 1 | single | 1 | 147 | 86 | 170 | 85 | 1 | 85 | 1 | 1 | 0 |
|  | 1 | single | 1 | 144 | 75 | 144 | 74 | 1 | 70 | 1 | 1 | 0 |
|  | 1 | single | 1 | 144 | 63 | 120 | 59 | 4 | 61 | 2 | 1 | 1 |
|  | 1 | single | 1 | 192 | 88 | 162 | 81 | 7 | 81 | 1 | 1 | 0 |
|  | 1 | single | 1 | 171 | 88 | 154 | 74 | 14 | 80 | 1 | 1 | 0 |
|  | 1 | single | 1 | 209 | 88 | 159 | 81 | 7 | 78 | 1 | 1 | 0 |
|  | 1 | single | 1 | 171 | 78 | 149 | 73 | 5 | 76 | 1 | 1 | 0 |
|  | 1 | single | 1 | 153 | 86 | 158 | 79 | 7 | 79 | 1 | 1 | 0 |
|  | 1 | single | 1 | 139 | 79 | 145 | 70 | 9 | 75 | 1 | 1 | 0 |
|  | 1 | single | 1 | 184 | 98 | 184 | 89 | 9 | 95 | 2 | 1 | 1 |
|  |  | Median Values: | | 171 | 86 | 158 | 79,0 | 7,0 | 79,0 | 1,0 | 1,0 | 0,0 |
|  |  | Mean Value: | | 167 | 84,45 | 157,36 | 78,0 | 6,5 | 79,4 | 1,2 | 1,0 | 0,2 |

Table 9 Results Summary 2D Sensor Data Processing - Scenario 1 for Sensor 4*

| Dataset Information | | | | | Sensor 4 | | | | | | | |
| --- | --- | --- | --- | --- | --- | --- | --- | --- | --- | --- | --- | --- |
|  |  |  |  |  | 2D Pose Estimation | | 2D Person Matching | | | 2D Person Tracking | | |
| Dataset Number | Scenario Number | Scenario Mode | Number Subjects | Frames | Open Pose | Alpha Pose | Matched | Unmachted OpenPose | Unmatched AlphaPose | Total | Valid | Invalid |
|  | 1 | single | 1 | 178 | 128 | 186 | 126 | 2 | 60 | 2 | 1 | 1 |
|  | 1 | single | 1 | 147 | 122 | 187 | 121 | 1 | 66 | 1 | 1 | 0 |
|  | 1 | single | 1 | 144 | 143 | 192 | 143 | 0 | 49 | 1 | 1 | 0 |
|  | 1 | single | 1 | 144 | 143 | 149 | 143 | 0 | 6 | 1 | 1 | 0 |
|  | 1 | single | 1 | 192 | 140 | 134 | 129 | 11 | 5 | 1 | 1 | 0 |
|  | 1 | single | 1 | 171 | 133 | 222 | 131 | 2 | 91 | 1 | 1 | 0 |
|  | 1 | single | 1 | 209 | 97 | 107 | 97 | 0 | 10 | 1 | 1 | 0 |
|  | 1 | single | 1 | 171 | 158 | 253 | 142 | 16 | 111 | 4 | 1 | 3 |
|  | 1 | single | 1 | 153 | 120 | 165 | 118 | 2 | 46 | 1 | 1 | 0 |
|  | 1 | single | 1 | 139 | 78 | 156 | 74 | 4 | 82 | 1 | 1 | 0 |
|  | 1 | single | 1 | 184 | 157 | 230 | 135 | 22 | 95 | 1 | 1 | 0 |
|  |  | Median Values: | | 171 | 133 | 186 | 129,0 | 2,0 | 60,0 | 1,0 | 1,0 | 0,0 |
|  |  | Mean Value: | | 167 | 129 | 180,09 | 123,5 | 5,5 | 56,5 | 1,4 | 1,0 | 0,4 |

* Each row of the table represents the summarized evaluation of a data set. The individual cells represent the cumulative number of input and output data of a processing step. The unit of the cell values is the number of objects processed during the data set's application. At the end of the table, the mean and average of each column are calculated for comparison against the other scenarios.

Table 10 Results Summary 3D Sensor Data Processing - Scenario 1*

| Dataset Information | | | | | 3D Multi Sensor Fusion | | | | | 3D Multi Sensor Tool | | | |
| --- | --- | --- | --- | --- | --- | --- | --- | --- | --- | --- | --- | --- | --- |
|  |  |  |  |  | 3D Person Matching | | 3D Person Tracking | | | Reference | | | |
| Dataset Number | Scenario Number | Scenario Mode | Number Subjects | Frames | In | Fused | Total | Valid | Invalid | Raw | Filtered | Person | NonPerson |
|  | 1 | single | 1 | 178 | 277 | 96 | 2 | 1 | 1 | 1 | 1 | 1 | 0 |
|  | 1 | single | 1 | 147 | 279 | 86 | 2 | 1 | 1 | 2 | 1 | 1 | 0 |
|  | 1 | single | 1 | 144 | 270 | 74 | 3 | 1 | 2 | 1 | 1 | 1 | 0 |
|  | 1 | single | 1 | 144 | 201 | 74 | 7 | 2 | 5 | 4 | 2 | 2 | 0 |
|  | 1 | single | 1 | 192 | 188 | 80 | 2 | 1 | 1 | 1 | 1 | 1 | 0 |
|  | 1 | single | 1 | 171 | 208 | 83 | 4 | 2 | 2 | 2 | 1 | 1 | 0 |
|  | 1 | single | 1 | 209 | 223 | 80 | 3 | 1 | 2 | 1 | 1 | 1 | 0 |
|  | 1 | single | 1 | 171 | 166 | 69 | 10 | 1 | 9 | 1 | 1 | 1 | 0 |
|  | 1 | single | 1 | 153 | 199 | 75 | 5 | 2 | 3 | 1 | 1 | 1 | 0 |
|  | 1 | single | 1 | 139 | 141 | 69 | 3 | 2 | 1 | 1 | 1 | 1 | 0 |
|  | 1 | single | 1 | 184 | 251 | 91 | 3 | 2 | 1 | 1 | 1 | 1 | 0 |
|  |  | Median Values: | | 171,0 | 208,0 | 80,0 | 3,0 | 1,0 | 2,0 | 1,0 | 1,0 | 1,0 | 0,0 |
|  |  | Mean Value: | | 166,5 | 218,5 | 79,7 | 4,0 | 1,5 | 2,5 | 1,5 | 1,1 | 1,1 | 0,0 |

* Each row of the table represents the summarized evaluation of a data set. The individual cells represent the cumulative number of input and output data of a processing step. The unit of the cell values is the number of objects processed during the data set's application. At the end of the table, the mean and average of each column are calculated for comparison against the other scenarios.

# APPENDIX II – Final statistical Evaluation Summary

Table 11 Evaluation Summary all Datasets and all Scenarios - Part 2.*

| Dataset Information | | | | | Sensor | Sensor | | | | |
| --- | --- | --- | --- | --- | --- | --- | --- | --- | --- | --- |
|  |  |  |  |  |  | 2D Pose Estimation | | 2D Pose Matching | | |
| Scenario Number | Number of Datasets | Scenario Mode | Number Subjects | Frames |  | Open Pose | Alpha Pose | Matched | Unmachted OpenPose | Unmatched AlphaPose |
| Scenario 1 | 11 | single | 1 | 167 | 1 | 312 | 459 | 293 | 19 | 166 |
|  |  |  |  |  | 2 | 252 | 368 | 239 | 13 | 128 |
|  |  |  |  |  | 3 | 84 | 157 | 78 | 6 | 79 |
|  |  |  |  |  | 4 | 129 | 180 | 124 | 5 | 56 |
| Scenario 2 | 10 | single | 1 | 182 | 1 | 294 | 509 | 284 | 10 | 180 |
|  |  |  |  |  | 2 | 193 | 273 | 181 | 12 | 95 |
|  |  |  |  |  | 3 | 93 | 170 | 83 | 7 | 79 |
|  |  |  |  |  | 4 | 121 | 158 | 108 | 13 | 50 |
| Scenario 3 | 10 | single | 1 | 227 | 1 | 354 | 587 | 347 | 7 | 240 |
|  |  |  |  |  | 2 | 257 | 325 | 216 | 41 | 109 |
|  |  |  |  |  | 3 | 120 | 204 | 110 | 8 | 96 |
|  |  |  |  |  | 4 | 161 | 196 | 142 | 19 | 54 |
| Scenario 4 | 9 | multi | 2 | 179 | 1 | 362 | 529 | 346 | 17 | 183 |
|  |  |  |  |  | 2 | 380 | 541 | 368 | 12 | 173 |
|  |  |  |  |  | 3 | 277 | 365 | 258 | 19 | 107 |
|  |  |  |  |  | 4 | 163 | 196 | 143 | 21 | 54 |
| Scenario 5 | 8 | multi | 2 | 203 | 1 | 412 | 564 | 381 | 30 | 183 |
|  |  |  |  |  | 2 | 430 | 618 | 405 | 25 | 214 |
|  |  |  |  |  | 3 | 187 | 269 | 176 | 11 | 93 |
|  |  |  |  |  | 4 | 310 | 424 | 289 | 21 | 136 |
| Scenario 6 | 8 | multi | 2 | 259 | 1 | 511 | 689 | 479 | 32 | 210 |
|  |  |  |  |  | 2 | 608 | 780 | 568 | 41 | 212 |
|  |  |  |  |  | 3 | 253 | 371 | 244 | 9 | 127 |
|  |  |  |  |  | 4 | 389 | 444 | 332 | 57 | 112 |

* The table summarizes the calculated average values for the individual evaluations of the scenarios. The cells contain the average number of objects for each processing step.

Table 12 Evaluation Summary all Datasets and all Scenarios - Part 2.*

| Dataset Information | Sensor | Sensor | | | 3D Multi Sensor Fusion | | | | | 3D Multi Sensor Tool | | | |
| --- | --- | --- | --- | --- | --- | --- | --- | --- | --- | --- | --- | --- | --- |
|  |  | 2D Person Tracking | | | 3D Person Matching | | 3D Person Tracking | | | Reference | | | |
| Scenario Number |  | Total | Valid | Invalid | In | Fused | Total | Valid | Invalid | Raw | Filtered | Person | Non  Person |
| Scenario 1 | 1 | 4 | 3 | 1 | 218,5 | 79,7 | 4 | 1 | 3 | 1 | 1 | 1 | 0 |
|  | 2 | 7 | 3 | 4 |  |  |  |  |  |  |  |  |  |
|  | 3 | 1 | 1 | 0 |  |  |  |  |  |  |  |  |  |
|  | 4 | 1 | 1 | 0 |  |  |  |  |  |  |  |  |  |
| Scenario 2 | 1 | 4 | 2 | 2 | 228,6 | 89,5 | 4 | 1 | 3 | 5 | 2 | 1 | 0 |
|  | 2 | 2 | 1 | 1 |  |  |  |  |  |  |  |  |  |
|  | 3 | 1 | 1 | 0 |  |  |  |  |  |  |  |  |  |
|  | 4 | 3 | 1 | 2 |  |  |  |  |  |  |  |  |  |
| Scenario 3 | 1 | 5 | 2 | 3 | 349,1 | 127,3 | 8 | 2 | 7 | 5 | 2 | 1 | 1 |
|  | 2 | 3 | 1 | 2 |  |  |  |  |  |  |  |  |  |
|  | 3 | 1 | 1 | 0 |  |  |  |  |  |  |  |  |  |
|  | 4 | 2 | 1 | 1 |  |  |  |  |  |  |  |  |  |
| Scenario 4 | 1 | 9 | 4 | 5 | 435,6 | 165,9 | 9 | 3 | 6 | 3 | 2 | 2 | 0 |
|  | 2 | 3 | 3 | 1 |  |  |  |  |  |  |  |  |  |
|  | 3 | 5 | 3 | 2 |  |  |  |  |  |  |  |  |  |
|  | 4 | 2 | 1 | 1 |  |  |  |  |  |  |  |  |  |
| Scenario 5 | 1 | 10 | 4 | 6 | 526,5 | 176,9 | 10 | 4 | 7 | 5 | 3 | 2 | 0 |
|  | 2 | 3 | 3 | 1 |  |  |  |  |  |  |  |  |  |
|  | 3 | 3 | 2 | 1 |  |  |  |  |  |  |  |  |  |
|  | 4 | 5 | 3 | 2 |  |  |  |  |  |  |  |  |  |
| Scenario 6 | 1 | 11 | 4 | 7 | 717,3 | 261,6 | 14 | 4 | 10 | 8 | 3 | 2 | 2 |
|  | 2 | 7 | 3 | 4 |  |  |  |  |  |  |  |  |  |
|  | 3 | 2 | 2 | 0 |  |  |  |  |  |  |  |  |  |
|  | 4 | 8 | 4 | 5 |  |  |  |  |  |  |  |  |  |

* The table summarizes the calculated average values for the individual evaluations of the scenarios. The cells contain the average number of objects for each processing step.
